# Association between timing and duration of breech presentation during pregnancy and developmental dysplasia of the hip: A case-control study

**DOI:** 10.1177/13674935211042198

**Published:** 2021-09-02

**Authors:** Annemieke Konijnendijk, Ellen Vrugteveen, Brenda Voorthuis, Magda Boere-Boonekamp

**Affiliations:** 1 Department of Biomedical Signals and Systems, 3230 University of Twente, Enschede, The Netherlands; 2 Department of Health Technology and Services Research, 3230 University of Twente, Enschede, The Netherlands

**Keywords:** Breech presentation, case control study, congenital hip dysplasia, epidemiology, pregnancy

## Abstract

This case-control study investigated the association between timing and duration of breech presentation in pregnancy and developmental dysplasia of the hip (DDH). Children with DDH aged 3 years or younger (*n* = 191) were compared with healthy controls (*n* = 209). Data on outcome, exposure and, covariates were collected using a parents’ self-report online questionnaire. Term children with breech presentation at one or more check-ups after 30.0 weeks gestation had a twofold higher risk of developing DDH compared to children who had never presented in breech (OR 2.01; 95% CI [1.28, 3.15]). The strength of the association increased with duration of breech presentation (5–8 weeks: OR 2.65; 95% CI [1.36, 5.18]; 9–12 weeks: OR 3.63; 95% CI [1.82, 7.24]). Children who had presented in breech at least once in gestational period 37.0-birth had a 3.24 (95% CI [1.86, 5.65]) times higher risk of DDH, whereas the risk for children with breech presentation in gestational period 30.0–36.6 only was not increased. Also after adjusting for confounders, children who had presented in breech after gestational week 37.0-birth had a more than threefold higher risk of DDH (OR 3.33; 95% CI [1.81, 6.13]) compared to children who were never in breech or in gestational period 30.0–36.6 only.

## Introduction

Developmental dysplasia of the hip (DDH) comprises a spectrum of disorders of the developing infant hip including hips that are unstable (dislocatable or dislocated) at birth and dysplastic hips with or without (sub) luxation when the child grows older (Shaw et al., 2016). The incidence of DDH varies greatly between countries and continents, but is estimated at one to 34 cases of DDH/1000 live births worldwide (Shorter et al., 2011; Zamborsky, 2019). Variation is related to whether and how (timing, method) population screening has been implemented, but also to factors like consanguinity rate and child care habits (tight swaddling). Risk factors for DDH reported by two meta-analyses include female gender, positive family history of DDH, and breech presentation in pregnancy and/or at birth (De Hundt et al., 2012; Ortiz-Neira et al., 2012). Evidence on increased risk of DDH for firstborn children (Ortiz-Neira et al., 2012) and for children with clicking hips at clinical examination (De Hundt et al., 2012) is conflicting. No significant increase of risk was found for prematurity, multiple gestation pregnancy, and mode of delivery (De Hundt et al., 2012; Ortiz-Neira et al., 2012).

Early screening and treatment prevent long term hip dysplasia and arthritis with complaints like impaired walking and pain in hip, knee, and lower back, requiring hip replacement (Shorter et al., 2011). Screening programs for DDH usually involve clinical examination in the neonatal period and during well-child consultations, ultrasound examination (universal or selective) or a combination of both (Shaw et al., 2016; Shorter et al., 2011). Besides family history, breech presentation in the third trimester of pregnancy has been included in many screening guidelines as an indication for ultrasonography (e.g., Boere-Boonekamp et al., 2018; Shaw et al., 2016). At lower gestational age, for example 29 weeks, 22% of fetuses are in breech presentation, but most fetuses turn spontaneously into cephalic position, resulting in four to five percent breech presentations at term (Cammu et al., 2014). Persistent breech presentation toward end of pregnancy is supposed to be unfavorable for hip development as it causes mechanical constraints to the hips (Shaw et al., 2016; Vaquero-Picado et al., 2019). Two meta-analyses present an increased risk of breech presentation for DDH with odds ratios (ORs) of 3.8 and 5.7 (De Hundt et al., 2012; Ortiz-Neira et al., 2012). However, neither these analyses nor the included studies give details on timing (which gestational weeks) and duration (number of weeks) of the breech presentation. Whether infants who were in breech for a longer period or who were in breech at the end of pregnancy are at similar risk of DDH compared to infants who were shorter in breech or only at earlier gestational periods is not known. This information is however essential for DDH screening guidelines. Based on the mechanical constraint theory, risk of DDH is expected to be higher in case of breech presentation near the end of pregnancy and with longer duration of breech presentation.

Current screening guidelines with the risk factor “breech presentation in the third trimester” cause large numbers of referrals of (asymptomatic) children for ultrasonography. If the definition of breech presentation and its association with DDH could be more specified, referral guidelines could be adapted, potentially leading to a more cost-effective screening.

### Aim

The aim is to investigate if children who were in breech presentation during pregnancy have a higher risk of DDH compared to children who were in cephalic presentation, and if so, if the association depends on duration and timing of the breech presentation.

## Methods

### Study design

A case-control study design was adopted. Parents of DDH cases and parents of control children filled out a questionnaire about their child’s presentation during pregnancy and at delivery (exposure) and a set of other well-known risk factors for DDH (covariates).

### Setting

In the Netherlands, the current screening program for DDH comprises physical examinations of all children at age 1, 3 and 6 months by a child healthcare physician in a well-baby clinic and additional ultrasound of children with abnormalities at hip examination and/or identified risk factors. Risk factors include positive family history of DDH or hip arthritis before the age of 45 years and breech presentation after 32.0 weeks gestation and/or at delivery (Boere-Boonekamp et al., 2018).

### Population

The case group consisted of children with diagnosed DDH, including dysplastic hips, subluxated hips, and luxated hips. The control group were children without DDH. Inclusion criteria were: younger than 3 years of age at time of data collection, born between July 2014 and May 2017, and with one or more known fetal presentation(s). Exclusion criterion was: child with DDH that is known to be part of a syndrome (Noordin et al., 2010). Informed consent was obtained from parents of all children.

Cases were all prevalent cases. Their parents were recruited through the “Patient association for abnormal hip development” (in Dutch: Vereniging Afwijkende Heupontwikkeling (VAH)). The association approached parents of potential cases by spreading an e-mail message with a link to a digital questionnaire to its members who had declared to be parents of a young child with DDH. In addition, they posted a message on their public Facebook page and in two closed groups on Facebook. Reminders were sent and posted after 1 week (Facebook) and 2 weeks (e-mail).

Parents of controls were recruited with the help of parents of cases. Cases’ parents were asked to request up to 3 parents (friends, acquaintances, colleagues) of a child without DDH younger than 3 years of age, to be willing to participate with their child as control. For this purpose, case parents could share the link of the questionnaire by social media or e-mail. Parents of cases were instructed not to ask family members of the child for whom they filled in the questionnaire.

### Variables

#### Outcome

At the beginning of the questionnaire, parents identified their child as case or control. Subsequently, parents of cases filled out additional questions about which hip was affected (left, right, both) and severity of the DDH (dysplasia, subluxation, or luxation).

#### Exposure

Breech presentation was defined as the child having been positioned head-up in the woman’s uterus, so with buttocks and none/one/both feet pointing toward the birth canal. Breech presentation at birth: the child lying in breech presentation in the week before birth. Breech presentation during pregnancy: the child lying in breech presentation at ≥1 check-ups in gestational period 30.0 till birth.

The fetus’ presentation was registered for gestational periods 30.0–31.6, 32.0–33.6, 34.0–35.6, 36.0–37.6, 38.0–38.6, 39.0–39.6, 40.0–40.6, 41.0–41.6, or 42.0–42.6 as: cephalic (head down), breech (head up), transverse, no information, or already gave birth. Pregnancy weeks were defined according to internationally accepted nomenclature (e.g., week 31 is gestational age 30.0–30.6). Transverse presentation (registered at least once in 39 children (9%)) was counted as “cephalic presentation” as literature on an association with DDH could not be found and it is not to be expected to cause mechanical constraint to the fetal hip (Shaw et al., 2016; Vaquero-Picado et al., 2019).

Two new variables, duration and timing of breech presentation, were created based on recorded data at check-ups in gestational period 30.0 until birth. Duration of breech presentation was divided in four categories: never; at all check-ups in 1–4 consecutive weeks; at all check-ups in 5–8 consecutive weeks; and at all check-ups in 9–13 consecutive weeks. Timing of breech presentation was divided in three categories: never; breech at ≥1 check-ups in gestational period 30.0–36.6 only; and breech at ≥1 check-ups in gestational period 37.0-birth (independent of previous positions).

#### Covariates

Background characteristics included child’s date of birth, age of diagnosis of DDH (in months), and zip code of the parents’ address. The following risk factors were measured: gender (male; female); (pre)maturity, i.e. born <37.0 (preterm) or ≥37.0 (term) gestational age; single/multiple birth; birth order (firstborn; consecutive child); delivery mode (vaginal; cesarean section); family history of DDH (negative; positive first degree i.e., diagnosed or suspected DDH before the age of 45 years in at least a first degree relative: parents and/or siblings; positive second degree i.e., diagnosed or suspected DDH before the age of 45 years in grandparents and/or uncles or aunts; not known, for example a donor parent).

### Data sources

Data of cases and controls on outcome, exposure, and covariates were collected among parents of cases and controls in an identical way, using a self-report questionnaire which was digitalized in LimeSurvey version 2.06+ (LimeSurvey Project Team and Schmitz, 2015).

Parents were asked to copy data on their child’s presentation, registered during antenatal check-ups on the pregnancy card, in the questionnaire. In the Netherlands, every woman goes for routine antenatal care to a (community or clinical) midwife or obstetrician, usually once every 2 weeks in gestational period 30.0–36.6 and once a week in gestational period 37.0 until birth.

### Data-analysis

#### Statistical methods

Data were imported into IBM SPSS Statistics version 23 (IBM Corp, 2015). First, for comparing cases and controls on background characteristics, and for comparing controls with the Dutch population, chi-squared tests were used.

Second, crude ORs with 95% confidence intervals (CIs) were calculated for associations between DDH and risk factors (Online Supplementary Appendix) and for associations between DDH and breech presentation at ≥1 check-ups in gestational period 30.0 till birth, breech presentation at birth, duration and timing of breech presentation (only term born children). *P*-values of the Pearson’s chi-square test were calculated, and in case of an expected cell count of less than five, *p*-values of the Fisher’s exact test were calculated.

Third, multivariable logistic regression analysis was performed to evaluate the association between DDH (yes/no) and exposure variable “timing of breech presentation,” adjusted for potential confounders (gender, single/multiple birth, birth order, family history in first degree). Results were expressed in adjusted ORs and corresponding 95% CIs.

The aim to include at least 150 cases in the multivariable logistic regression analysis was based on the rule of thumb of 10–20 cases for each independent variable (5–7 expected) (Vittinghoff and McCulloch, 2007).

All tests were two-sided, and *p*-values of <0.05 were considered statistically significant.

#### Imputation of missing data

In total, 68 (20.5%) parents noted at least one unknown presentation in their pregnancies. Since listwise deletion of these cases would lead to the loss of a vast amount of data, and many plausible predictors for the unknown presentations were present in the data, in case the child’s presentation at an antenatal check-up was unknown, this data was imputed. Reasons for missing data were: no check-up in that specific week; fetus’ presentation was or could not be determined, was not registered or was not communicated with the pregnant woman. The number of unknown presentations at check-ups was 31, 36, 21, 20, 7, 6, 1, 1, and 1 in gestational periods 30.0–31.6, 32.0–33.6, 34.0–35.6, 36.0–36.6, 37.0–37.6, 38.0–38.6, 39.0–39.6, 40.0–40.6, and 41.0–41.6, respectively.

Chi-square tests between child’s presentation in each pregnancy week and gender, multiple birth, birth order, and family history in first degree showed no significant associations. Therefore, it was assumed that data were missing completely at random (MCAR) (Little and Rubin, 2019). Five missing data imputations were created through multiple imputation using chained equations (MICEs) with a polytomous logistic regression method. For each pregnancy week, variables with a Cramér’s V over 0.4 were included as predictor variables. Imputation was conducted in R version 3.6.1 (R Core Team, 2019) using version 3.6.0 of the mice package (Buuren and Groothuis-Oudshoorn, 2010). Pooled ORs were calculated using Rubin’s rules. A pooled $\chi^{2}$-statistic for the multivariable model was calculated using the formula by Enders (Enders, 2010).

#### Ethical approval

This research was approved by the institutional ethical committee of the faculty of Behavioral, Management, and Social Sciences of the University of Twente (Reference number 17224).

## Results

### Participants

Parents of 191 DDH cases and 209 control children responded to the questionnaire. Of these 400 respondents, 46 (11.5%) were excluded because of the following reasons: child older than 3 years or date of birth in the future (*n* = 41; 10.3%), arthrogryposis multiplex congenita (*n* = 2; 0.5%), and no information on the child’s presentations during pregnancy (*n* = 3; 0.8%). Thus, the study population consisted of 161 cases and 193 controls. Cases were originating from all 12 provinces and controls from 11 provinces in the Netherlands.

Of the 161 cases, 66 (41.0%) had left-sided DDH, 28 (17.4%) right-sided DDH, and 67 (41.6%) bilateral DDH. The 133 left-sided DDH hips included 74 (55.6%) hips with dysplasia only, 8 (6.0%) subluxated hips and 49 (36.8%) luxated hips; in 2 (1.5%) hips, the severity of DDH was unknown. The 95 right-sided DDH hips included 67 (70.5%) hips with dysplasia only, five (5.3%) subluxated hips and 22 (23.2%) luxated hips; in one (1.1%) hip, the severity of DDH was unknown.

The group of 193 control children was representative of the Dutch population for gender (male: 52.8% vs. 51.5% in population), single/multiple birth (multiple birth: 1.6% vs. 1.5% in population), birth order (firstborn: 48.2% vs. 45.3% in population) (Centraal Bureau voor de Statistiek, 2017a), and maturity (preterm: 9.3% vs. 6.7% in population) (Centraal Bureau voor de Statistiek, 2017b), but had fewer vaginal births than expected (77.7% vs. 83.3%; *p* = .036) (MacFarlane et al., 2016).

Compared to controls, DDH cases were significantly more often female (82.0% vs. 47.2%; OR 5.10; 95% CI [3.21, 8.34]), less often preterm (3.1% vs. 9.3%; OR 0.31; 95% CI [0.11,0.86]), more often firstborn (62.1% vs. 48.2%; OR 1.76; 95% CI [1.15, 2.70]), less often born vaginally (59.0% vs. 77.7%; OR 0.41; 95% CI [0.26, 0.66]), and had more often a positive family history in one or more first degree relatives (29.2% vs. 10.4%; OR 3.85; 95% CI [2.15, 6.90]). Online Supplementary Appendix 1 provides an overview of the prevalence of risk factors in cases and controls.

### Main results

For analysis of the association between DDH and characteristics of the breech presentation, preterm infants were excluded (five cases and 18 controls; Tables 1 and 2). Table 1 presents the univariate analyses with crude ORs (and 95% CIs).

**Table 1.** Breech characteristics of DDH cases and control children: breech presentation at least at one check-up, presentation at birth, duration, and timing of breech presentation (only term born children).

Variable	Cases *n* = 156 *n* (%)	Controls *n* = 175 *n* (%)	Crude OR [95% CI]
Breech presentation at ≥1 check-ups in gestational period 30.0-birth			
No	76 (48.5)	114 (65.4)	Ref
Yes	80 (51.5)	61 (34.6)	2.01 [1.28, 3.15]
Presentation at birth			
Other	101 (64.7)	154 (88.0)	Ref
Breech	55 (35.3)	21 (12.0)	3.99 [2.28, 7.01]
Duration of breech presentation			
Never	76 (48.5)	114 (65.4)	Ref
1–4 weeks	145 (9.4)	28 (16.1)	.78 [0.39, 1.58]
5–8 weeks	32 (20.6)	18 (10.5)	2.65 [1.36, 5.18]
9–13 weeks	34 (21.5)	14 (8.0)	3.63 [1.82, 7.24]
Timing of breech presentation			
Never in breech	76 (48.5)	114 (65.4)	Ref
Breech at ≥1 check-ups in gestational period 30.0–36.6 only	26 (16.9)	35 (20.2)	1.13 [.62, 2.07]
Breech at ≥1 check-ups in gestational period 37.0-birth (independent of previous positions)	54 (34.6)	25 (14.4)	3.24 [1.86, 5.65]

OR = odds ratio.

**Table 2.** Multivariable logistic regression of the association between DDH and the timing of breech presentation in pregnancy (in gestational period 37.0 till birth versus never or in gestational period 30.0–36.6 only) in term born children (156 cases, 175 controls).

	B (SE)	Odds ratio	95% CI for odds ratio	
			Lower	Upper
No DDH versus DDH				
Breech presentation in gestational period 37.0-birth^a^	1.20 (.31)*	3.33	1.81	6.13
Female gender	1.84 (.28)*	6.26	3.60	10.92
Multiple birth	.87 (1.29)	2.38	.65	8.68
Firstborn	.44 (.26)	1.55	.93	2.58
Positive family history first degree relative	1.36 (.34)*	3.89	2.78	5.45

Note. R^2^ = .24 (Cox & Snell), .32 (Nagelkerke). D_2_ = 17.97, F (5, 86142827.86) = 17.97, *p* < .001. *p < .001.

^a^Breech presentation at one or more check-ups in gestational period 37.0-birth, independent of previous presentations.

Term children with a breech presentation recorded at least during one check-up after 30.0 weeks gestation had a 2.01 (95% CI [1.28, 3.15]) times higher risk of DDH compared to children with no breech presentations recorded during that period. Children presenting in breech at birth had an almost 4 times higher risk (OR 3.99; 95% CI [2.28, 7.01]). The increased risk of DDH was higher with longer duration of breech presentation; 5–8 weeks: 2.65 (95% CI [1.36, 5.18]; 9–13 weeks: 3.63 (95% CI [1.82, 7.24]). Children with breech presentation in gestational period 37.0-birth during at least one check-up had a 3.24 (95% CI [1.86, 5.65]) times higher risk of DDH compared to children who were never in breech, whereas breech presentation only in gestational period 30.0–36.6 did not increase the risk of DDH.

After adjustment for gender, single/multiple birth, birth order, and positive family history in first degree, logistic regression analysis showed a 3.33 times higher risk of DDH (95% CI [1.81, 6.13]) for children who had presented in breech in gestational period 37.0-birth compared to children who were never in breech or in gestational period 30.0–36.6 only (Table 2).

## Discussion

### Main results

This case-control study, comparing children with and without DDH, shows that term children with breech presentation at one or more check-ups after 30 weeks gestation had on average a twofold higher risk of developing DDH. The longer the breech presentation was present, the higher the risk of DDH. After adjustment for potential confounders, children with breech presentation in the last pregnancy weeks had a more than three times higher risk of DDH than children who were never in breech or in gestational period 30.0–36.6 only.

### Interpretation

Compared to our results (OR 3.33; 95% CI [1.81, 6.13]), De Hundt et al. (2012) found a stronger association of “breech presentation” with DDH (OR 5.7; 95% CI [4.4, 7.4]), and Ortiz-Neira et al. (2012) found a similar association of “breech presentation during pregnancy and at delivery” with DDH (RR 3.75; 95% CI [2.25, 6.24]). However, both studies do not report details on the exact criteria for the definition of breech exposure that they used to include articles in their meta-analyses.

Apparently, experiencing breech presentation at some point in pregnancy is quite common. In our study, in more than half (51.5%) of DDH cases a breech presentation was recorded during at least one antenatal check-up in gestational period 30.0 until birth. The percentage of control children who had ever had a breech presentation is also rather high (34.6%). Unfortunately, this figure cannot be compared with other studies as these report prevalence rates (ranging from 13–20% at 30 weeks gestation to 4–5% at term) which are based on cross-sectional data (Cammu et al., 2014; Fox and Chapman, 2006; Hill, 1990). Due to its high prevalence, application of the risk factor “breech presentation in gestational period 30.0 till birth” in selective US screening programs (e.g., in the US (Shaw et al., 2016) and the Netherlands (Boere-Boonekamp et al., 2018)) inevitably leads to a high number of hip ultrasounds with, on average, a low positive predictive value.

Our finding that, in term children, the increased risk of DDH only applies to those who presented in breech in gestational period 37.0-birth (more than threefold increase) favors limiting the breech criterion as indication for selective US to the last pregnancy weeks (37.0-birth). Both this finding and the finding that the risk of DDH increases with longer duration of the breech presentation are consistent with the mechanical constraint theory (Vaquero-Picado et al., 2019). Toward the end of pregnancy, sustained hamstring forces on the hip (e.g., as a result of prolonged knee extension in frank breech presentation), the maternal pelvis acting on the fetus’ hip joints, and the rapid growth rate of the fetus create increased mechanical forces that may impair hip development in breech presenting infants (Hegde et al., 2020; Lambeek et al., 2013; Vaquero-Picado et al., 2019). The mechanical constraint theory is also supported by the finding of Lambeek et al. (2013) that children born in cephalic presentation after a successful external cephalic version (ECV) at 34 weeks gestation have a lower incidence of DDH (2.8%) compared with children born in breech presentation after unsuccessful ECV (9.3%). Lambeek et al. (2013) reason that successful ECV prevents the development of DDH because these fetuses do not descend into the pelvis in breech presentation during the last part of pregnancy, which may protect them against increasing mechanical forces acting on the hips. However, according to the authors the possibility of reverse causation cannot be ruled out, that is, that as a consequence of malfunction in the hip and different leg function in utero resulting from DDH, these children are more likely to remain in breech presentation.

Based on our study, we conclude that, in general, preterm infants have a lower risk of DDH compared to term infants (OR 0.31; 95% CI [0.11,0.86]), but we cannot draw conclusions on the DDH risk of preterm infants that present in breech at delivery. Hegde et al. (2020) examined a large group of preterm and term breech born infants and found that both groups have a similar incidence of DDH (diagnosed with ultrasound (Graf IIC) or on X-ray: 12.2% in preterm infants, 11.5% in term infants). This finding does not fit with our conclusion that only breech presentation in the last weeks of pregnancy is a risk factor for DDH in term children. Although Hedge et al. (2020) mention that preterm infants are exposed to less intrauterine forces and for a shorter duration, they do not provide an explanation for their findings not being consistent with the mechanical constraint theory (Vaquero-Picado et al., 2019).

### Generalizability

In our study, cases were all member of the patients’ association for abnormal hip development (VAH). Therefore, they most probably represent the more serious DDH cases in the Netherlands, where incidence rates of 0.4% for hip dislocation and 3.7% for all types of DDH in the general population of newborns were reported (Boere-Boonekamp et al.,1998). That our case group is a relatively more severely affected group is confirmed by the high percentage of (sub)luxations among the cases. However, as to our knowledge, severity of DDH does not moderate the association between breech position and DDH, we do not expect our results to be influenced by the overrepresentation of serious DDH cases in our sample. The group of control children was representative of the Dutch population for all measured background characteristics, except for delivery mode (less vaginal births in control group). We therefore expect the results of this study to be generalizable to the Dutch population and to populations in comparable western countries. However, caution is advised as the sample size of cases and controls is relatively small, the numbers in some subcategories are low, and only the most important background characteristics were measured.

### Limitations

Our study had several limitations. First, control children were recruited by convenience sampling. To avoid selection bias, we gave clear instructions to parents of DDH cases on inviting parents of control children. Nevertheless, the number of term breech children in the last week of pregnancy in our control group (*n* = 21; 12.0%) proved to be higher than the average number of four to five percent reported for a general population of term born infants (Cammu et al., 2014). Possibly, since we informed parents about the aim of our study (i.e., to investigate the association between breech presentation and DDH), more parents of (control) children with a breech presentation decided to participate in the study. This means that the association between breech presentation in the last pregnancy weeks and DDH may be higher than our findings suggest. Second, in case no pregnancy card was available (*n* = 89; 55.3% in case group, *n* = 127; 65.8% in control group), parents filled out questions on their child’s presentation based on their recollection which may have caused information bias. To minimize the risk of including parents not remembering their child’s presentation in pregnancy, we set the inclusion criterion at children younger than 3 years. Third, in 30 (18.6%) DDH cases and 38 (19.7%) control children, one or more presentations during pregnancy check-ups were unknown. Imputation of missing data, allowed because data were missing at random, prevented us from having to exclude roughly 20% of the participants that made an effort to complete our questionnaire and generated valuable data.

### Implications for practice

The new evidence gathered in this study can be used to improve clinical guidelines on screening for DDH in countries that have chosen to implement the combination of clinical screening and selective ultrasound screening. If our findings are confirmed in other studies with larger samples and prospectively recorded data on the fetus’ presentation, referral of children for an ultrasound screening examination because of increased risk of DDH resulting from breech presentation can be limited to those children who presented in breech after gestational week 37.0. The modified definition would help child healthcare physicians in well-baby clinics to correctly identify the children with breech presentation that need a hip ultrasound. The number of referrals would decrease, referrals would have a higher positive predictive value, and cost-effectiveness of screening would probably increase.

## Conclusion

Of term children with breech presentation, only those with breech presentation from gestational week 37.0 onward have an increased risk (more than threefold) of DDH. The risk of DDH increases with the length of the breech presentation. We recommend a large population-based study using data of obstetric (exposure) and orthopedic (outcome) records to confirm our findings on the association of breech presentation and DDH.

## Supplemental Material

sj-pdf-1-chc-10.1177_13674935211042198 – Supplemental Material for Association between timing and duration of breech presentation during pregnancy and developmental dysplasia of the hip: A case-control study Click here for additional data file. Supplemental Material, sj-pdf-1-chc-10.1177_13674935211042198 for Association between timing and duration of breech presentation during pregnancy and developmental dysplasia of the hip: A case-control study by Annemieke Konijnendijk, Ellen Vrugteveen, Brenda Voorthuis and Magda Boere-Boonekamp in Journal of Child Health Care
