# Supplementary material for: Association between timing and duration of breech presentation during pregnancy and developmental dysplasia of the hip: A case-control study
Source: J Child Health Care. 2021 Sep 2;27(1):35–45. doi: 10.1177/13674935211042198 (PMC9932609; doi:10.1177/13674935211042198)
Supplement: sj-pdf-1-chc-10.1177_13674935211042198 – Supplemental Material for Association between timing and duration of breech presentation during pregnancy and developmental dysplasia of the hip: A case-control study [file sj-pdf-1-chc-10.1177_13674935211042198.pdf]

Appendix 1. Prevalence of risk factors, not related to presentation in pregnancy and at birth, in the 161 DDH cases and 193 controls.

Variable	Cases n = 161 n (%)	Controls n = 193 n (%)	Crude OR (95% CI)
Gender			
Boy	29 (18.0)	102 (52.8)	ref
Girl	132 (82.0)	91 (47.2)	5.10 (3.21 – 8.34)
(Pre)maturity			
Term (≥ gestational age 37.0)	156 (96.9)	175 (90.7)	ref
Preterm (< 37.0)	5 (3.1)	18 (9.3)	0.31 (0.11 – 0.86)
Single/multiple birth			
Single	159 (98.8)	190 (98.4)	ref
Multiple	2 (1.2)	3 (1.6)	0.79 (0.13 – 4.80)
Birth order			
Consecutive child	61 (37.9)	100 (51.8)	ref
Firstborn	100 (62.1)	93 (48.2)	1.76 (1.15 – 2.70)
Delivery mode			
Cesarean section	66 (41.0)	43 (22.3)	ref
Vaginal	95 (59.0)	150 (77.7)	0.41 (0.26 – 0.66)
Family history			
Negative	94 (58.4)	154 (79.8)	ref
Positive, 1st degree family relative(s)	47 (29.2)	20 (10.4)	3.85 (2.15 – 6.90)
Positive, only 2nd degree family relative(s)	20 (12.4)	19 (9.8)	1.73 (0.88 – 3.40)

OR = Odds ratio
